# Effect of Dietary Modification on Gastric Mucosa, Gastrointestinal Symptoms and Nutritional Status of Patients With Early Gastric Cancer After Endoscopic Submucosal Dissection Surgery: A Retrospective Cohort Study

**DOI:** 10.3389/fnut.2022.741630

**Published:** 2022-03-22

**Authors:** Yebing Zhang, Chengxia Liu, Xingbin Ma, Lei Xu, Xiuhua Wang, Xin Wang, Jingrun Cao, Aiguo Ma, Tao Gao

**Affiliations:** ^1^Institute of Nutrition and Health, School of Public Health, Qingdao University, Qingdao, China; ^2^Binzhou Medical University Hospital, Binzhou, China; ^3^Huai’an Center for Disease Control and Prevention, Huai’an, China

**Keywords:** early gastric cancer, endoscopic submucosal dissection, dietary pattern, dietary modification, cohort study

## Abstract

Food is an important factor affecting the treatment of patients with early gastric cancer (EGC). We have established a hospital cohort to guide dietary patterns and observe the health status of patients with EGC after endoscopic submucosal dissection (ESD) after dietary modification. A total of 273 patients with EGC who underwent ESD were recruited to the cohort. They were given dietary instruction and education through a dietary manual and were followed up for 12 months. If the dietary pattern changed to the “traditional food” pattern (high consumption of vegetables, wheat products, and red meat) after the nutritional guidance, subjects were defined as the improvement diet group. Dietary patterns focused on “alcohol and fish” (drink a lot of wine and beer and eating freshwater and marine fish) or “coarse cereals” (mainly whole grains, beans and poultry) were the main ones in the unimproved diet group. The nutritional status, gastric mucosa, and gastrointestinal symptoms of the two groups of patients before and after the dietary instruction were compared. Compared with the unimproved diet group, the endoscopic performance score and the symptom score in the improved diet group were decreased by an average of 1.31 and 1.90, respectively. Except for lymphocyte count (*P* = 0.227), total protein (*P* < 0.000), albumin (*P* = 0.003), globulin (*P* = 0.014), red blood cell count (*P* < 0.000), and hemoglobin (*P* < 0.000) values were improved to varying degrees. After changing the diet, the intake of wheat products and vegetables in the improved diet group increased by 15.58 and 17.52%, respectively, while the intake of alcohol, fish, and pickled products was reduced by 43.36, 36.43, and 31.41%, respectively. After 1 year of dietary adjustment, the nutritional status, gastric mucosa, and gastrointestinal symptoms of patients with EGC after ESD eating the "traditional food" diet were all improved.

## Introduction

**FIGURE 1:**
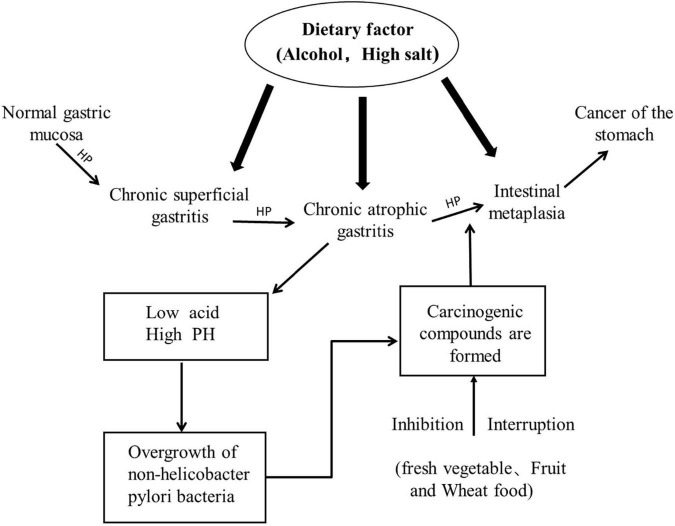
Carcinogenesis pattern of intestinal type gastric cancer.

Gastric cancer ranks fifth among malignant tumors globally, accounting for approximately 5.6% of cancers, and it is the third leading cause of cancer-related deaths (1). The occurrence of gastric cancer is a multi-step and multi-factor process, resulting from the complex interaction between genetic susceptibility and environmental factors. Early gastric cancer (EGC) refers to gastric cancer tissue confined to the mucosa and submucosa, regardless of its size or lymph node metastasis status (2), and it is mainly manifested as intestinal gastric cancer (Figure 1). The long-term prognosis of patients with EGC is good, and the 5-year survival rate can reach 90 to 95% (3). Endoscopic submucosal dissection (ESD) is a surgical treatment commonly used in recent years in patients with EGC, and it can completely remove EGC at one time. However, after eradication of *Helicobacter pylori* in EGC patients after ESD, metachronous gastric cancer can still occur, with an incidence of 2.4% (4). Early studies have shown that gastric mucosal inflammation, atrophy, and intestinal metaplasia are independent risk factors (5) for metachronous cancer that can be controlled by diet (6–8). In addition to the damage of gastric mucosa, we can also observe the recovery of gastric environment in patients with EGC through gastrointestinal symptoms. Gastrointestinal symptoms are mostly caused by peptic ulcer and malignant lesions (9, 10). The relief of symptoms (such as vomiting, nausea, and acid regurgitation) is related to specific carbohydrates, proteins and fats or micronutrients (11). Early gastric cancer is in the precancerous stage of gastric cancer, and it is best to be treated during this period, whether through surgery, drugs, diet, or lifestyle changes (12), so we proposed to further study the dietary pattern suitable for gastric cancer patients after early ESD surgery.

In 2018, we conducted a cross-sectional study on the dietary patterns of patients with atrophic gastritis (13) and obtained three typical dietary patterns through principal component analysis (PCA). The first is "alcohol and fish," including high consumption of wine, beer, and freshwater and marine fish. The second is "traditional food," which means eating a lot of vegetables, wheat products, and red meat. The third type is "coarse cereals," which means eating large amounts of whole grains, beans and poultry. In this study, "alcohol and fish" and "coarse cereals" were negatively correlated with atrophic gastritis, while "traditional food" was not correlated with atrophic gastritis. Gastric atrophy and intestinal metaplasia of the gastric mucosa are collectively referred to as chronic atrophic gastritis and are considered precancerous lesions (14). Based on this research, we decided to explore a dietary pattern suitable for EGC patients after ESD. In 2007, the World Cancer Research Foundation/American Cancer Institute (WCRF/AICR) noted, "Non-starchy vegetables, especially onion vegetables and fruits can prevent gastric cancer" (15). In this report, a daily intake of 50 grams of allium vegetables was associated with a 23% reduction in the risk of gastric cancer. A meta-analysis involving 11 case-control and cohort studies showed that the higher the salt intake, the risk of GC increased by 22% (16). However, people’s daily intake consists of a variety of foods. The resulting complex combination of dietary components is likely to produce interactive or synergistic effects (17, 18). In 2021, a case-control study (19) of 415 people in South Korea found that a diet including high amounts of vegetables and fruits is associated with a reduction in GC risk. In a retrospective cohort study in Ireland (20), a low FODMAP (fermentable oligosaccharides, disaccharides, monosaccharides, and polyols) diet was used by patients with irritable bowel syndrome (IBS) for 1 year, which significantly improved the symptoms of IBS. Therefore, the dietary pattern formed by the combination of foods may have a greater impact on certain diseases than the individual foods.

Existing research mainly focuses on the relationship between certain single nutrients or foods and the incidence of gastric cancer (21–23). Research on dietary patterns and EGC is still lacking. Therefore, through this cohort study, we hope to explore the dietary patterns suitable for EGC patients after ESD that will relieve postoperative symptoms and promote the recovery of the gastric environment, as well as add more research evidence for future dietary guidelines.

## Materials and Methods

### Ethics Approval

The study was approved by the Ethics Committee of the Affiliated Hospital of Binzhou Medical University and was complied with the ethical guidelines of the Declaration of Helsinki. All participants have obtained informed consent (Clinical trial registration number: ChiCTR2100045405; Ethical certification number: KYLL-2021-01).

### Subjects

From January 2019 to December 2020, a total of 273 patients with EGC who underwent ESD were recruited to the hospital-based cohort study in the Affiliated Hospital of Binzhou Medical College located in Binzhou City, China. The hospital is the Yellow River Delta Regional Medical Center. It is the only tertiary A hospital in the region that independently conducts ESD. In February 2018, it was rated as a demonstration base for early gastrointestinal tumor screening in Shandong Province.

The inclusion criteria were as follows: (1) Between 18 and 66 years old, gender not limited; (2) Endoscopic diagnosis of EGC, surrounding mucosal background shows gastric mucosal atrophy; (3) ESD complete resection of the lesion, the pathology high-grade intraepithelial neoplasia or highly or moderately differentiated adenocarcinoma, no residual horizontal margin, vertical invasion depth of 500 μm, no vascular metastasis, no distant metastasis; (4) Except for the dietary pattern, there was no significant change in lifestyle; and (5) Voluntary participation and signed informed consent. The exclusion criteria were (1) History of gastrointestinal surgery, malignant tumor, or severe neurological disease; (2) Positive for *H. pylori* before ESD treatment; (3) Pregnancy or lactation; and (4) Long-term (>3 months) use of omeprazole or another gastric mucosa protector.

### Design

According to the results of a cross-sectional study in 2018 (24), a dietary guidebook for patients who had ESD after EGC was designed by a clinical registered dietitian certified by the Chinese Dietitian Association. The dietary guidelines included: (1) The dietary pattern after EGC surgery, giving priority to eating "traditional food" and reducing "alcohol and fish" and "coarse grains;" (2) Introduction of the intake of energy, protein, carbohydrates, and micronutrients and the type of food intake; and (3) A reminder to record food intake and existing problems regularly so that the follow-up calls can proceed smoothly. We conducted a telephone return visit every 4 months to monitor the patient’s dietary improvement and digestive symptoms and make detailed records. The patient was notified to come to the hospital for review on the 12th month after surgery. According to the follow-up situation, the included population was divided into two groups. If the 4, 8, and 12th month follow-ups after nutritional guidance all answered “traditional food” mode, they were defined as the improved diet group, and patients who adopted the "traditional food" mode before dietary guidance were excluded. At the 4, 8, and 12th months of follow-up, those who adopted the “alcohol and fish” or “coarse grain” model were defined as the unimproved diet group. In addition, the basic data, biochemical data, and endoscopy reports of patients before and 12 months after the instruction were collected, and the diet survey and clinical symptom survey were carried out.

### Survey

The questionnaire survey included general information (such as age, gender, income, education level, etc.), history of chronic diseases, family history of gastric cancer and history of HP infection. The dietary survey was conducted with a food frequency table and a 3-day 24-hour diet review table. The food frequency table was used to understand the nutritional intake of patients, and the 3-day 24-hour diet review table was used to check the patient’s eating pattern. The food frequency questionnaire (FFQ) consists of 74 foods, including grains (rice, wheat foods, and whole grains), potatoes, beans, vegetables, fruits, dairy products, eggs, meats (pork, beef, and lamb) poultry, fish, preserved products, alcohol, and tea (25). The frequency items included g/daily, g/weekly, g/monthly, g/yearly, and finally converted into the daily intake (3). Physical examination included height and weight, all measured with an electronic scale. Body mass index (BMI) was calculated and according to the classification criteria of the Chinese population, was categorized as thin (BMI <18.5 kg/m^2^), normal (BMI 18.5∼23.9 kg/m^2^), overweight (BMI 24.0∼27.9 kg/m^2^), and obese (BMI >28 kg/m^2^).

### Indicators

(1) Endoscopic performance score: according to the Kyoto gastritis standard classification (26), we scored the gastroscopic performance before and at 12 months after the dietary instruction (see Table 1.1 for the scoring criteria).

**TABLE 1.1:** Endoscopic performance scoring criteria for gastric cancer risk.

Feature	Scoring criteria
Shrinking	0 points (no C-0∼C-1); 1 point (mild C-2∼C-3); 2 points (severe O-1∼O-P)
Intestinal metaplasia	0 points (no); 1 point (gastric antrum); 2 points (gastric antrum, gastric body)
Fold enlargement	0 points (no); 1 point (yes)
Chicken skin	0 points (no); 1 point (yes)
Diffuse redness	0 points (no); 1 point (mild, RAC+ in some areas); 2 points (severe)

(2) Digestive tract symptom score: Any uncomfortable reaction (including abdominal pain, hiccups, bloating, burning sensation, acid reflux, and eating less) was defined as an uncomfortable reaction. The daily stomach discomfort reaction was recorded through the questionnaire, and the stomach discomfort reaction was scored before and after the dietary instruction (see Table 1.2 for the digestive tract symptom score).

**TABLE 1.2:** Gastrointestinal symptom score table.

Clinical symptoms	Score			
	0	1	2	3
Stomach ache	No	Mild, short duration, no medication required	Moderate, pain for a long time, more than 4 h a day, still tolerable	Severe, severe pain, continuous, need medication to relieve
Belching	No	Seizures	Mild attack, causing discomfort in both ribs	Frequent attacks, causing pain in ribs
Bloating	No	Abdominal distension is worse in a short time	Abdominal bloating is severe and does not subside for a long time	Bloating all day long
Burning sensation	No	Occasionally and short-lived	Appear frequently	Persistent discomfort
Acid reflux	No	Occasionally and short-lived (Occasionally spit acid)	1 to 2 times a day (Eat discomfort or vomit acid)	Acid reflux several times in a day (Frequent spitting of acid)
Eat less	No	Poor appetite, less than 1/3 of appetite reduction	Poor appetite, reduced appetite by 1/3-1/2	There is no appetite, and the appetite is reduced by more than 2/3

(3) Biochemical data: we collected the total protein (TP), albumin (Alb), hemoglobin (Hb), red blood cell count (RBC) and other data of patients with EGC to reflect the changes in the nutritional status of the patients before and after dietary guidance. At the same time, we also determined the incidence of hypoalbuminemia and low hemoglobin in the two groups after 1 year of follow-up. The reference values of each index are: TP 60∼80 g/L, Alb-40∼55 g/L, Hb-120∼160 g/L (male),110∼150 g/L (female). An Alb content less than 35 g/L was considered hypoalbuminemia. Low Hb in males was considered to be less than 120 g/L and less than 110 g/L in females (27).

## Statistical Analysis

Data were analyzed using SPSS 24.0 software. We compared the pre-instruction and 12-month data of the improved diet group and the unimproved diet group. Quantitative data such as age conforming to a normal distribution or an approximately normal distribution were statistically analyzed by an independent sample *t*-test. The Wilcoxon signed-rank test was used to analyze non-normal data such as dietary intake, endoscopic performance score, symptom score, and biochemical indicators. The qualitative data such as gender, hypertension, family history, etc., of the two groups were all tested by the χ2 test. Fisher’s accurate probability method was used to compare qualitative data such as education level, income, diabetes, and BMI. The above analysis is a two-sided test, and the test result shows a statistical difference when *P* < 0.05.

## Results

### Basic Situation

**FIGURE 2:**
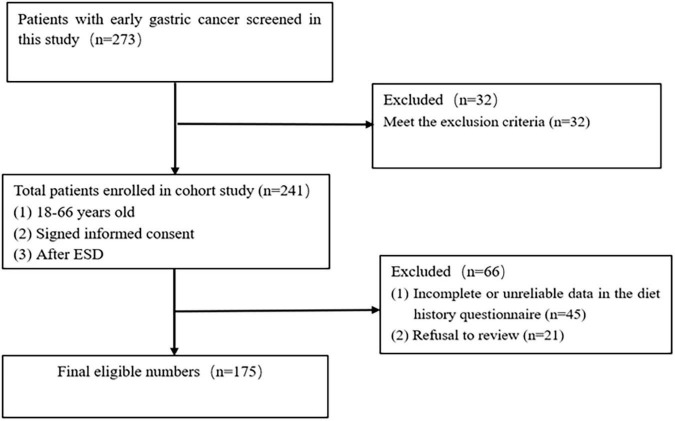
Flow chart of the study population.

The flowchart of the study population is shown in Figure 2. A total of 273 patients joined the study, and 98 potential participants were excluded for the following reasons: (1) Refusal to review (*n* = 21); (2) Met the exclusion criteria (*n* = 32); (3) Incomplete or unreliable data in the diet history questionnaire (*n* = 45). The study finally included 175 participants.

We paired the baseline data and related indicators before the intervention, including age (*P* = 0.629), gender (*P* = 0.921), educational level (*P* = 0.636), income (*P* = 0.286), hypertension (*P* = 0.422), diabetes (*P* = 1.00), family history (*P* = 0.099), history of HP infection (*P* = 0.575), body mass index (*P* = 0.350), etc., and these were not found to be statistically different (Table 2).

**TABLE 2:** Demographic data of research subjects.

Basic Information	Grouping	Improved diet group (*n* = 140)	Unimproved diet group (*n* = 35)	*P*
Age		61.66 ± 9.11	60.80 ± 10.75	0.629
Gender	Male	115 (82.1)	29 (82.9)	0.921
	Female	25 (17.9)	6 (17.1)	
Education level	Elementary school and below	44 (31.4)	15 (42.9)	0.636
	Junior high school	55 (39.3)	11 (31.4)	
	High school and technical Secondary school	33 (23.6)	7 (20.0)	
	Junior college	6 (4.3)	1 (2.9)	
	Bachelor degree and above	2 (1.4)	1 (2.9)	
Income	<2000	50(35.7)	17 (48.6)	0.286
	2000–5000	49 (35.0)	8 (22.9)	
	5000–8000	32 (22.9)	6 (17.1)	
	>8000	9 (6.4)	4 (11.4)	
Hypertension	Yes	31 (22.1)	10 (28.6)	0.422
	No	109(77.9)	25 (71.4)	
Diabetes	Yes	13 (9.3)	3 (8.6)	1.00
	No	127 (90.7)	32 (91.4)	
Family history	Yes	93 (66.4)	18 (51.4)	0.099
	No	47 (33.6)	17 (48.6)	
History of HP infection	Yes	72 (51.4)	16 (54.3)	0.575
	No	68 (48.6)	19 (45.7)	
BMI	<18.5 kg/m^2^	3 (1.1)	2 (5.7)	0.350
	18.5–23.9 kg/m^2^	65 (23.2)	12 (34.3)	
	24.0–27.9 kg/m^2^	54 (19.3)	15 (42.9)	
	>28 kg/m^2^	18 (6.4)	6 (17.1)	

### Endoscopic Manifestations and Stomach Symptoms

**FIGURE 3:**
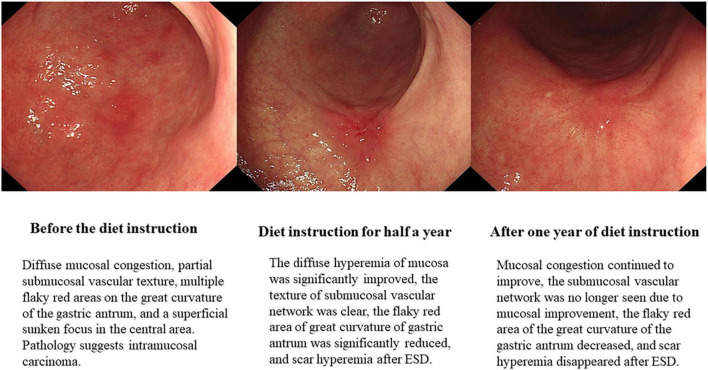
Changes of gastric mucosa in patients with early gastric cancer before and after dietary guidance.

According to the endoscopic performance scoring standard of the risk of gastric cancer in the Kyoto global consensus report on *Helicobacter pylori* gastritis (26), the average score of the diet improvement group before instruction was 4.82, and the average score after 12 months of instruction was 3.50 (*P* < 0.000); The average score of the unimproved diet group before instruction was 5.25, and the average score after 12 months of instruction was 4.80 (*P* = 0.008). The difference in endoscopic performance scores between the two groups before and after dietary guidance was statistically significant (Table 3). The score difference between the two groups was analyzed by the Mann–Whitney U test. The endoscopic performance score of the diet improvement group was reduced by an average of 1.31, and the score for diet unimproved group was reduced by an average of 0.45. The difference in scores was statistically significant (*P* < 0.000) (Table 4). At the same time, we also provided the gastric endoscopic images of patients with EGC before dietary guidance, half a year and 1 year, which showed a significant improvement in the gastric environment (Figure 3).

**TABLE 3:** Comparison of endoscopic performance scores and symptom scores between the diet improvement group and the diet non-improvement group before and after dietary guidance.

	Improved diet group (*n* = 140)		*P*	Unimproved diet group (*n* = 35)		*P*
	Before dietary guidance	1 year after guidance		Before dietary guidance	1 year after guidance	
Endoscopic performance	4.82 ± 1.54	3.50 ± 1.29	<0.000	5.25 ± 1.31	4.80 ± 1.15	0.008
Digestive symptoms	2.27 ± 1.31	0.36 ± 0.64	<0.000	2.51 ± 1.50	1.17 ± 0.82	<0.000

**TABLE 4:** Comparison of the difference between the endoscopic performance score and the symptom score of the diet improvement group and the diet non-improvement group at 1 year after dietary guidance.

	Improved diet group (*n* = 140)	Unimproved diet group (*n* = 35)	*P*
Endoscopic score	−1.31 ± 1.09	−0.45 ± 0.88	<0.000
Digestive symptom score	−1.90 ± 1.32	−1.34 ± 1.18	0.022

Scores are assigned according to the research subjects’ digestive tract symptoms and severity, and the scoring criteria are shown in the Methods section. The results showed that there was a statistical difference in symptom scores between the improved diet group (*P* < 0.000) and the unimproved diet group (*P* < 0.000) before and after dietary guidance (Table 3). After 12 months of dietary guidance, the symptom score of the improved diet group dropped by an average of 1.90 and in the unimproved group dropped by an average of 1.34. The score difference between the two groups was statistically significant (*P* = 0.022) (Table 4).

### Nutritional Status

**FIGURE 4:**
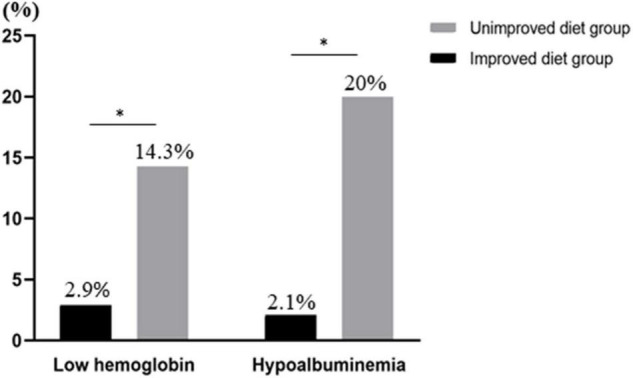
Comparison of low hemoglobin and hypoalbuminemia between the diet-improved group and the diet-unimproved group (*P < 0.05).

The 175 subjects were divided into two groups, 140 people in the improved diet group and 35 people in the unimproved diet group. We compared the TP, Alb, Hb, and RBC of the two groups to determine whether the nutritional status of the patients improved after 1 year of dietary guidance. The results showed that the related indicators of the patients in the improved diet group improved before and after the instruction: TP (*P* = 0.001), Alb (*P* < 0.000), Hb (*P* = 0.019), and RBC (*P* < 0.000). We compared the differences between the improvement group and the unimproved group’s nutritional indicators before and after changes, and TP (*P* < 0.000), Alb (*P* = 0.003), Hb (*P* < 0.000), and RBC (*P* < 0.000) were statistically different. After adopting the “traditional food” diet, the nutritional status of patients improved, but nutritional status of patients who adhered to the "alcohol and fish" or "coarse cereals" did not (Tables 5, 6). In addition, we also found that after 1 year of follow up, there were differences in the incidence of hypoalbuminemia (*P* = 0.001) and low hemoglobin (*P* = 0.017), and the incidence in the improved diet group was significantly lower than that in the non-improved diet group (Figure 4).

**TABLE 5:** Comparison of nutritional status before and after guidance between the diet improvement group and the diet non-improvement group.

Measure	Improved diet group (*n* = 140)		*P*	Unimproved diet group (*n* = 35)		*P*
	Before dietary guidance	1 year after guidance		Before dietary guidance	1 year after guidance	
TP	68.74 ± 5.30	70.89 ± 4.91	0.001	69.43 ± 4.91	67.56 ± 5.12	0.069
Alb	41.49 ± 4.95	43.39 ± 3.20	<0.000	42.57 ± 3.85	41.77 ± 3.52	0.264
RBC	4.55 ± 0.56	4.77 ± 0.45	<0.000	4.79 ± 0.43	4.51 ± 0.47	0.002
Hb	137.97 ± 15.02	146.14 ± 13.81	<0.000	147.11 ± 12.69	139.91 ± 17.84	0.007

*TP, total protein; Alb, albumin; RBC, red blood count; Hb, hemoglobin.*

**TABLE 6:** Comparison of nutritional status difference between the two groups after 1 year.

Species	Improved diet group (*n* = 140)	Unimproved diet group (*n* = 35)	*P*
TP	2.38 ± 4.60	−1.86 ± 5.35	<0.000
Alb	2.00 ± 4.99	−0.80 ± 4.18	0.003
RBC	0.21 ± 0.47	−0.27 ± 0.48	<0.000
Hb	8.17 ± 11.72	−7.20 ± 14.79	<0.000

*TP, total protein; Alb, albumin; RBC, red blood count; Hb, hemoglobin.*

### Diet

We collected the dietary questionnaires before the patient’s dietary guidelines and the 12th month of dietary guidance. After analysis, we found that the diet improvement group that adopted the "traditional food" model increased intake of wheat products by 15.58% and vegetables by 17.52%. Alcohol, fish, and pickled food intake decreased by 43.36, 36.43, and 31.41%, respectively. Changing the dietary intake shows that adopting the “traditional food” model can help improve the nutritional status, gastric mucosa, and gastrointestinal symptoms of patients with EGC after ESD (Table 7).

**TABLE 7:** Dietary changes in the diet improvement group and the diet non-improvement group.

Species	Improved diet group (*n* = 140)		*P*	Unimproved diet group (*n* = 35)		*P*
	Before dietary intervention	1 year after intervention		Before dietary intervention	1 year after intervention	
Rice (g/d)	88.95 ± 35.14	99.68 ± 35.02	0.009	107.14 ± 45.60	88.57 ± 38.03	0.075
Coarse grains (g/d)	31.08 ± 41.96	40.53 ± 53.34	0.104	45.00 ± 46.88	65.72 ± 59.13	0.150
Pasta (g/d)	99.86 ± 47.64	120.9 ± 42.05	0.000	100.71 ± 26.06	97.90 ± 14.11	0.578
Tubers (g/d)	89.37 ± 54.21	103.47 ± 49.87	0.012	94.51 ± 51.20	116.15 ± 68.68	0.211
Beans (g/d)	79.02 ± 61.37	86.19 ± 58.97	0.343	83.05 ± 56.88	128.58 ± 59.74	0.002
Red meat (g/d)	58.03 ± 39.99	40.19 ± 34.76	0.000	54.28 ± 31.20	48.14 ± 48.26	0.533
Poultry (g/d)	99.10 ± 77.06	67.10 ± 66.96	0.000	115.71 ± 94.53	105.71 ± 71.25	0.611
Fish (g/d)	60.25 ± 91.90	38.30 ± 52.54	0.013	117.14 ± 98.47	93.57 ± 96.12	0.261
Vegetables (g/d)	120.25 ± 49.70	145.46 ± 43.98	0.000	137.85 ± 51.58	123.05 ± 54.43	0.358
Fruit (g/d)	117.14 ± 96.29	106.78 ± 76.50	0.332	105.00 ± 69.08	99.28 ± 78.70	0.672
Pickled products (g/d)	48.73 ± 43.67	33.42 ± 46.48	0.004	41.48 ± 40.77	56.74 ± 34.40	0.066
Milk and eggs (g/d)	145.96 ± 132.99	180.98 ± 187.68	0.056	146.17 ± 142.93	153.14 ± 157.44	0.836
Alcohol (g/d)	65.89 ± 133.58	37.32 ± 94.28	0.049	60.71 ± 94.18	67.85 ± 121.20	0.796
Tea (g/d)	45.57 ± 63.74	27.96 ± 48.60	0.007	17.42 ± 25.87	49.85 ± 59.11	0.005

## Discussion

The results of this study showed that 1 year after the diet was adjusted to “traditional food,” intake of the vegetable and wheat foods in the diet-improved group increased by 17.52 and 15.58%, respectively. It was observed that the patient healing was significantly improved. The nutritional status, gastric mucosa, and gastrointestinal symptoms of the improved diet group were statistically different from those of the unimproved diet group. The Alb (43.39 ± 3.20), TP (70.89 ± 4.91), Hb (146.14 ± 13.81), and RBC (4.77 ± 0.45) in patients with EGC were significantly higher than those in the unimproved diet group. The endoscopic performance score decreased by an average of 1.31, and the symptom score decreased by an average of 1.90.

The gastric mucosa plays an important role in maintaining the physiological functions of the stomach. As the barrier in the stomach, the gastric mucosa can protect deep tissues from gastric juice components and exogenous gastric mucosal irritants (28). A retrospective cohort study (29) consisting of 10,185 subjects found that drinking alcohol is an independent risk factor for mucosal atrophy. Alcohol can regulate the release of inflammatory factors, activate granulocytes, cause protease secretion, produce reactive oxygen species, and contract blood vessels, and enhanced vascular permeability can change gastric acid secretions and induce acute gastric mucosal damage (30, 31). In addition, pickled products and salted fish are sources of sodium nitrate and nitrite, which can react with amino acids in the stomach to form N-nitro compounds, which are called chemical gastric carcinogens (32). Nitrite causes epithelial cell damage, and high concentrations of sodium chloride cause mucosal damage, which in turn increases the sensitivity to mutagenesis or cancer (33–35). However, proper food intake can repair the gastric mucosa and reduce gastrointestinal symptoms. In two studies in South Korea (36) and Italy (37), it was found that eating high levels of vegetables and fruits is associated with lowering the risk of gastric cancer, possibly because fruits and vegetables are rich in antioxidants, which can protect the gastric mucosa. Avoiding inflammation and inhibiting endogenous nitrosation reduces the damage of *H. pylori* in the body (38, 39). In 2020, an animal experiment (40) showed that wheat peptides protect the gastric mucosa from the lesions caused by ethanol in rats by improving gastric microcirculation and inhibiting inflammation mediated by the nuclear factor (NF)-κB signal transduction pathway. Wheat peptides are extracted from natural food wheat flour, and then the small molecule peptide material is obtained by directed enzyme digestion and specific oligopeptide separation technology, it is abundant in wheat foods. In addition to the repair of the gastric mucosa, digestive symptoms were also reduced in the diet improvement group. It may be that the vitamins in fruits and vegetables promote gastrointestinal motility, reduce reflux, and appropriately increase high dietary fiber. The intake of foods such as fungi and oats can strengthen nutrition and increase esophageal sphincter pressure. Wheat-based foods are fine grains that can reduce the burden of gastrointestinal peristalsis in patients with EGC and relieve symptoms such as abdominal pain. To some extent, this explains that traditional food patterns will have a positive impact on patients with EGC.

Serum TP levels mainly reflect liver synthesis function and protein loss caused by kidney disease (41). Alb plays an important role in maintaining body fluid osmotic pressure, removing oxygen free radicals related to inflammatory diseases, and maintaining pH as a plasma buffer (42). The half-life of Alb is 21 days, which can reflect the condition of the body’s protein over a few weeks to several months (41). Low Hb (anemia) is a common nutritional problem in EGC (42). Protein malnutrition can inhibit the proliferation and differentiation of hematopoietic stem cells (43), inhibit immune responses, and cause chronic inflammation, leading to changes in iron metabolism (44) that result in decreased hemoglobin. These indicators can be used to evaluate the nutritional status of patients (45). Nutritional status is one of the important factors affecting the treatment of patients with EGC (46). Characteristics of EGC patients include weight loss, nutritional deficiencies, impaired immunity, and disordered inflammation levels (47). Malnutrition decreases cellular immunity, increases susceptibility to inflammation and infection, and weakens the body’s ability to repair itself, leading to aggravation of the disease (17). Postoperative patients with EGC may also have gastric mucosal damage caused by inflammation (48, 49) and gastrointestinal symptoms that affect appetite (50). Some patients do not understand how to choose suitable foods after surgery because of their limited education. These reasons can cause malnutrition in patients with EGC. Through dietary guidance to adjust the type of food intake, repair the gastric mucosa, and alleviate gastrointestinal symptoms, the nutritional status of patients can be improved.

The strengths of this study include the following: (1) According to the literature, this is the first study on EGC and diet patterns in China. (2) It provides specific dietary combinations and intake sources of carbohydrates, fats, proteins and vitamins, and is not limited to a single food; (3) It uses the authoritative International Kyoto Gastritis Standard Scale to evaluate the gastric mucosal state, and the results obtained thereby are more accurate. (4) It eliminates the interference of *H. pylori* and adjust factors such as age, gender, economic status, family genetic history and others; (5) The Affiliated Hospital of Binzhou Medical College is a regional medical center in the Yellow River Delta. It is the only hospital that independently conducts ESD. Most patients will choose to come to this hospital for review, which reduces the rate of loss to follow up during the research process. This study also has several limitations: (1) Because patients with positive *H. pylori* were excluded, the number of people included in this study was limited; (2) the FFQ used to conduct dietary surveys relies on the patient’s memory to answer, and there will be a certain information bias in this process, but at the same time we used a 3-day 24-hour diet review to reduce this bias.

## Conclusion

In this cohort study, 1 year after the diet model was changed from the "alcohol and fish" or "coarse grains" model to the "traditional food" model, nutritional status, gastric mucosa, and gastrointestinal symptoms of patients with EGC after ESD were improved. Improvement of diet is a major factor in optimizing the quality of life of these patients after surgery. However, whether changes in dietary patterns can reduce the incidence of metachronous cancer in EGC patients remains to be confirmed by further studies.

## Data Availability Statement

The raw data supporting the conclusions of this article will be made available by the authors, without undue reservation.

## Ethics Statement

The studies involving human participants were reviewed and approved by Ethics Committee of the Affiliated Hospital of Binzhou Medical University; Affiliated Hospital of Binzhou Medical College. The patients/participants provided their written informed consent to participate in this study. Written informed consent was obtained from the individual(s) for the publication of any potentially identifiable images or data included in this article.

## Author Contributions

AM and TG designed research and had primary responsibility for final content. YZ, XM, XUW, and JC conducted the research. YZ, LX, and XNW analyzed data. YZ and CL wrote the manuscript. All authors contributed to the article and approved the submitted version.

## Conflict of Interest

The authors declare that the research was conducted in the absence of any commercial or financial relationships that could be construed as a potential conflict of interest.

## Publisher’s Note

All claims expressed in this article are solely those of the authors and do not necessarily represent those of their affiliated organizations, or those of the publisher, the editors and the reviewers. Any product that may be evaluated in this article, or claim that may be made by its manufacturer, is not guaranteed or endorsed by the publisher.
